# Healthcare Engagement as a Potential Source of Psychological Distress among People without Religious Beliefs: A Systematic Review

**DOI:** 10.3390/healthcare5020019

**Published:** 2017-04-05

**Authors:** Samuel R. Weber, James W. Lomax, Kenneth I. Pargament

**Affiliations:** 1 Intermountain Healthcare, Logan, UT 84341, USA; 2 Department of Psychiatry, Baylor College of Medicine, Houston, TX 77030, USA; jlomax@bcm.edu; 3 Department of Psychology, Bowling Green State University, Bowling Green, OH 43403, USA; kpargam@bgsu.edu

**Keywords:** religion, atheism, agnosticism, mental health, psychological distress

## Abstract

Research into religion and mental health is increasing, but nonbelievers in terms of religion are often overlooked. Research has shown that nonbelievers experience various forms of psychological distress and that the negative perception of nonbelievers by others is a potential source of distress. This review builds on that research by identifying another potential source of psychological distress for nonbelievers: engagement with the healthcare system. Poor understanding of nonbelievers by healthcare professionals may lead to impaired communication in the healthcare setting, resulting in distress. Attempts by nonbelievers to avoid distress may result in different patterns of healthcare utilization. Awareness of these concerns may help healthcare providers to minimize distress among their nonbelieving patients.

## 1. Introduction

In recent years, greater numbers of studies have examined the link between religious belief and psychological well-being [1,2,3,4]. Despite this increased attention, many such studies overlook the psychological health of nonbelievers (i.e., atheists and agnostics). In a recent review, Weber et al. [5] found that various forms of psychological distress are experienced by nonbelievers. For example, nonbelievers are more likely than believers to struggle with anger toward God and difficulty forgiving God. In contrast, nonbelievers are just as well or better off than their believing counterparts in terms of responding to the death of a public religious figure, coping with the challenges of old age, and overall happiness. Conflicting evidence arises when nonbelievers are compared with believers for death anxiety and spiritual quality of life. Greater certainty in one’s belief system (religious or nonreligious) is associated with greater psychological health. Additionally, Weber et al. identified a potential source of distress for nonbelievers: the negative perception of nonbelievers by others. They suggested that additional sources of distress may be discovered with further research.

Research has shown that medical appointments and decisions regarding healthcare can be sources of psychological distress. For example, routine pelvic examinations for women have been shown to provoke anxiety in both patients and physicians [6]. Merely being in the presence of a physician is enough to produce the well-known “white coat effect,” an anxiety state which may lead to falsely elevated blood pressure readings [7]. Although patients’ psychological distress is known to be negatively associated with their perceived quality of communication with health providers, problems in doctor–patient communication are still very common [8].

Nonbelievers in terms of religion are a minority group in the United States [9], and as such their preferences may not be as well understood by healthcare professionals. A lack of understanding between nonbelieving patients and their providers may contribute to poor communication. If providers are unaware of the preferences unique to their nonbelieving patients, they may make insensitive remarks or recommendations for services in which nonbelievers have no interest. Poor communication in the healthcare setting may lead to increased distress for the patient, who may in turn avoid certain recommendations or seek a less distressing treatment setting. The objective of this study is to examine engagement with healthcare as a potential source of distress for nonbelievers.

## 2. Methods

**Figure 1:**
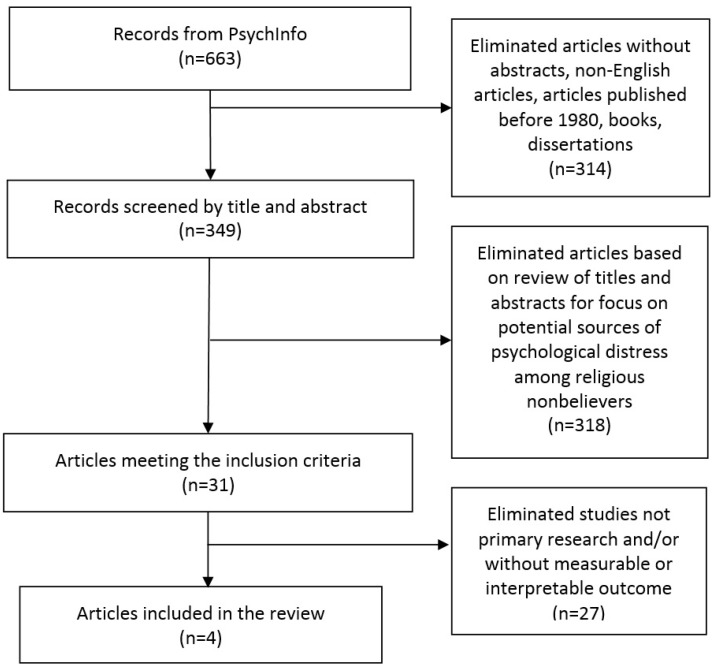
Flow diagram of study selection.

We searched the PsycINFO database using the following search terms: atheis*, agnosti*, apostasy, apostate, and deconversion. Given our expectation that there would be little extant literature on the topic, broad search terms were used to cast a wide net. The resultant group of articles was then further culled to limit articles to those with abstracts, those written in English, and those published no earlier than 1980, excluding books and dissertations. After reviewing all titles and abstracts, we selected articles that met our focus on healthcare engagement as a potential source of psychological distress among nonbelievers. Criteria for inclusion in this review were established as follows: studies were to be primary research and have some form of measurable or interpretable outcome. By mutual consensus, we eliminated articles from the pool that did not meet our inclusion criteria. We then examined the reference lists of the identified articles for possible additional articles for inclusion. Four articles were identified for inclusion in the present review (Figure 1).

## 3. Results

The results of our search are shown in Table 1. All four studies focused on engagement with healthcare among nonbelievers.

### 3.1. Definitions and Measurements of Lack of Religious Belief

Articles by Baker, Smith, and Tonigan defined a lack of religious belief in terms of religious affiliation, such as atheism, agnosticism, no religion, unsure, humanism, or rationalism [10,11,12]. Atheists were defined in Smith-Stoner’s study as people who do “not accept that there are any gods, heaven, hell, devils, souls, miracles, afterlife, or anything else supernatural” [13] (p. 923).

Measurement of nonbelief varied between studies. Articles from Baker and Smith measured nonbelief by means of a survey or questionnaire item identifying one’s religious affiliation as atheist, agnostic, or no religion [11,12]. Tonigan inquired after spiritual-based practices using the Religious Behavior and Background questionnaire (RBB) [10]. Membership in an atheist organization was used to gauge belief status in Smith-Stoner’s paper [13].

### 3.2. Healthcare Decision-Making as a Potential Source of Psychological Distress

All four articles pointed out potentially stressful modalities of healthcare and support that are avoided by nonbelievers. None of the participants in an online survey who identified as having no religious beliefs indicated that they would seek counseling services from a priest, rabbi, minister, or coven [11]. A sample of atheists and agnostics in the United Kingdom perceived religious forms of treatment for depression as ineffective [12]. Participants from a sample of 88 atheists expressed concern that healthcare workers or chaplains may attempt to proselytize them as part of their end of life care, and indicated that conversion attempts or prayer before death were not acceptable [13]. In a study of participation in Alcoholics Anonymous (AA), atheists and agnostics combined attended AA significantly less often than did believers over a 15 month period. Atheists had the highest rates of nonparticipation in AA, with 65.4% reporting no AA attendance for the entire 15 month period. Additionally, the highest proportion of AA disaffiliation after initiating treatment was found among agnostic clients (group mean scores for proportion AA attendance at 3 months: 0.15; at 6 months: 0.06). Patients with uncertain beliefs in terms of religion had the highest rates of drinking, consequences, and dependence severity prior to treatment, and showed the least amount of improvement in terms of abstinence during and after AA treatment [10].

The four studies in this review also reported less distressing healthcare options used preferentially by nonbelievers. From the Smith study, atheists and agnostics look to their friends and family for counseling and support more often than members of religious groups do [11]. Nonbelievers are no different from believers in their perceptions regarding the efficacy of social, cognitive/self-help, and professional/medical treatments for depression according to Baker [12]. Although nonbelievers are less likely to be referred to, initiate, and sustain participation in Alcoholics Anonymous, Tonigan showed that nonbelievers who do participate in AA nevertheless derive benefits comparable to believers [10]. According to Smith-Stoner, many atheists have clear preferences regarding end-of-life care, including pain and symptom management, clear decision-making, preparation for death, completion, and affirmation of the whole person. Additionally, 95% of the atheists sampled supported physician-assisted suicide, and many indicated that donating one’s organs or body to science was an important means of contributing to others through death [13].

## 4. Discussion

Engagement with the healthcare system is a potential source of psychological distress among nonbelievers, and attempts to avoid distress may result in different patterns of healthcare utilization compared to believers. Nonbelievers tend to avoid religious forms of care, but they clearly appreciate the support of qualified healthcare professionals [12] and are more likely than believers to seek support from friends and family [11]. Nonbelievers and believers perceive professional/medical, cognitive/self-help, and social treatments for depression similarly in terms of effectiveness [12]. Nonbelievers are less likely than believers to participate in Alcoholics Anonymous, although those who do participate derive similar benefit compared to believers [10]. This suggests that healthcare providers may do well to take a different approach when encouraging nonbelieving patients to participate in Alcoholics Anonymous, perhaps emphasizing increased abstinence rates and community support over spiritual themes. Atheists appear to have clear preferences for end of life care. It is unclear how well these preferences are respected, particularly in light of atheist concerns of being proselytized by their healthcare workers prior to death [13].

This review has some limitations. The primary limitation is the small number of studies that met criteria for inclusion. This is not surprising, given that the previous literature survey by Weber et al. [5] found only four studies that examined another potential source of psychological distress for nonbelievers: the negative perception of nonbelievers by others. This lends further evidence toward our assertion that nonbelievers have been overlooked in the scientific literature up to this point. It should be noted that the studies we selected were from dissimilar scientific disciplines, and two of the studies were conducted outside of the United States. There may be cultural or national issues that affect the applicability of these results, particularly in countries with religious demographics different from the United States. The literature itself has some limitations. Definitions of nonbelief were generally not detailed or thorough, and the measurements used were not consistent or standardized. Consistent definitions of nonbelief in future studies would help ensure that results are interpreted correctly. Smith-Stoner’s survey of end of life preferences sampled only atheists with no comparison group of believers. Such a comparison group would be valuable in examining belief-specific preferences versus general social trends. Although studies by Tonigan et al. and Baker and Cruickshank distinguished between atheists and agnostics, Smith and Simmonds lumped atheists and agnostics together into a “no religion” group. However, the needs and behaviors of atheists and agnostics are not identical. It may be appropriate to conceptualize atheists as certain in their nonbelief and agnostics as uncertain in their nonbelief. Although some agnostics may be certain in their preference for agnosticism over atheism, religious agnosticism nevertheless maintains a lack of certainty about the existence of God. These differences in certainty of belief may contribute to each group’s psychological well-being [5]. Additional psychological research into the experience of nonbelievers in healthcare settings compared to that of believers is needed to allow for more robust conclusions.

## 5. Conclusions

Religious belief is one component of individual psychological well-being. Past research has demonstrated that various forms of psychological distress are experienced by nonbelievers and that the negative perception of nonbelievers by others is a potential source of distress [5]. This study builds upon those results, showing that healthcare engagement is another potential source of distress for nonbelievers. With increasing interest in religion in the psychology literature, nonbelievers should not be overlooked. It is our hope that, with increased attention to nonbelief, healthcare workers may be better equipped to minimize distress for their nonbelieving patients.

## Figures and Tables

**Table 1:** Healthcare engagement among nonbelievers.

Author	Nonbelief Defined	Measurement Tools	Study Design	Results
**Tonigan 2002 [10]**	Self-identified religious affiliation: atheist, agnostic, unsure	Religious Behavior and Background (RBB) questionnaire Alcoholics Anonymous Involvement (AAI) self-report tool Form 90 interview for client drinking	USA Project MATCH outpatient and aftercare samples, *N* = 1526	**AA: benefits and likelihood of participation** God belief is unimportant in deriving AA-related benefit, but atheist and agnostic clients are less likely to initiate and sustain AA attendance. Unsure patients had the highest rates of drinking and the least improvement after treatment.
**Smith 2006 [11]**	“No religion” group: agnostic, atheist, no religion, humanist, or rationalist	Demographic/help-seeking questionnaire Paranormal Beliefs scale	Australian online study, *N* = 414	**Relationship between help-seeking and adherence to mainstream religion, alternative religion, and no religion** “No religion” group less likely to choose “priest/rabbi/minister/coven/etc.” for counseling support compared with mainstream and alternative groups. No religion group chose “friend or relative” to greater extent than either group.
**Baker 2010 [12]**	Self-reported religious affiliation: atheist, agnostic	63 item questionnaire: Demographics Depression-Happiness Scale (DHS) Salience Scale Measurement of frequency of religious practices Scale analyzing beliefs about treatments for depression	British study, November 2007–February 2008, *N* = 471 (130 atheists, 104 agnostics)	**Differences in perceived efficacy of depression treatment form** Social, cognitive/self-help, or professional/medical treatments: no significant difference between all groups Religious treatments: believers rated more highly than nonbelievers.
**Smith-Stoner 2007 [13]**	Atheist: members of two atheist organizations	Online and paper surveys	USA pilot study, members of two atheist organizations, 3 months in 2005, *N* = 88 (all atheists)	**Atheist preferences for end-of-life care** Include pain and symptom management, clear decision making, preparation for death, completion, and affirmation of the whole person (including respect for nonbelief), contributing to others (organ donation), and support of physician-assisted suicide. Atheists expressed concern that healthcare workers may attempt to proselytize them prior to death.
